# *GFP* as a marker for transient gene transfer and expression in *Mycoplasma hyorhinis*

**DOI:** 10.1186/s40064-016-2358-3

**Published:** 2016-06-17

**Authors:** Hassan Z. A. Ishag, Maojun Liu, Ruosong Yang, Qiyan Xiong, Zhixin Feng, Guoqing Shao

**Affiliations:** Institute of Veterinary Medicine, Jiangsu Academy of Agricultural Sciences, Key Laboratory of Veterinary Biological Engineering and Technology, Ministry of Agriculture, National Research Center for Engineering and Technology of Veterinary Bio-products, Nanjing, 210014 China; College of Veterinary Sciences, University of Nyala, Nyala, Sudan

**Keywords:** *Mycoplasma hyorhinis*, Plasmid, *GFP*, Expression

## Abstract

*Mycoplasma hyorhinis* (*M. hyorhinis*) is an opportunistic pathogen of pigs and has been shown to transform cell cultures, which has increased the interest of researchers. The green florescence proteins (*GFP*) gene of *Aquorea victoria*, proved to be a vital marker to identify transformed cells in mixed populations. Use of *GFP* to observe gene transfer and expression in *M. hyorhinis* (strain HUB-1) has not been described. We have constructed a pMD18-O/MHRgfp plasmid containing the *p97* gene promoter, origin of replication, tetracycline resistance marker and *GFP* gene controlled by the *p97* gene promoter. The plasmid transformed into *M. hyorhinis* with a frequency of ~4 × 10^−3^ cfu/µg plasmid DNA and could be detected by PCR amplification of the *GFP* gene from the total DNA of the transformant mycoplasmas. Analysis of a single clone grown on KM2-Agar containing tetracycline, showed a green fluorescence color. Conclusively, this report suggests the usefulness of *GFP* to monitor transient gene transfer and expression in *M. hyorhinis,* eventually minimizing screening procedures for gene transfer and expression.

*Mycoplasma hyorhinis* (*M. hyorhinis*) is a commensal pathogen of swine that also causes lung lesions and inflammation (Razin et al. 1998), and is thought to contribute to the development of cell transformation in vitro (Namiki et al. 2009). These properties of *M. hyorhinis* have increased interest to the researchers.

Whole genome sequence of *M. hyorhinis* strain HUB-1 was determined (Liu et al. 2010), and expression of foreign antigens in *M. hyorhinis* might help to produce recombinant engineered strains. However, a method based on *GFP* expressing plasmids to evaluate the transformation and expression of foreign genes in *M. hyorhinis* has not been described. Several methods to monitor gene activity in cells are available such as the formation of fusion proteins with coding sequences for *β*-galactosidase, firefly luciferase, and bacterial luciferase (Stewart and Williams 1992). But, these methods are of limited use since they require exogenous substrates or cofactors. The green florescence proteins (*GFP*) of jellyfish *Aequoreavictoria* is a unique tool to monitor gene transfer and expression (Cubitt et al. 1995). Using *GFP* might help to construct an efficient reporter system for *M. hyorhinis.* Here, we constructed a plasmid expressing *GFP* fluorescence and optimized conditions for transformation by electroporation.

*M. hyorhinis* strain HUB-1 (GenBank accession CP002170.1) was provided by Prof. Xiao Shaobo (Huazhong Agricultural University, China) and was grown at 37 °C in KM2 cell-free liquid medium (a modified Friis medium) containing 20 % (v/v) swine serum (Xiong et al. 2016). KM2-Agar was prepared by adding 0.7 % Agar (Biowest Agarose ^®^G-10; Gene Company Limited, Chi Wan, Hong Kong) to KM2 medium and was incubated at 37 °C to grow the visible colonies. Tetracycline hydrochloride (Sigma-Aldrich) was used at 0.01 μg/ml.

We previously constructed a plasmid pMD18-TOgfp encoding tetracycline resistance gene (*tetM*) controlled by the *p97* gene promoter, *GFP* gene also controlled by the *p97* gene promoter and *oriC* of *M. hyopneumoniae* attenuated strain *(*168L) (GenBank accession 507382422) (Ishag et al. 2016). The purpose of this plasmid was to express *GFP* in *M. hyopneumoniae* strain 168L. It is well known that, the *p97* gene functions as an adhesion molecule for *M. hyopneumoniae* and the activity of this promoter was previously described in *oriC*-plasmids of *M. hyopneumoniae* (Maglennon et al. 2013). Here, we further evaluated the potential of this promoter in *M. hyorhinis*.

The presence of the *oriC* in plasmids is necessary to maintain the plasmid in the host, and for mycoplasmas, the *oriC* has been found to be species specific (Cordova et al. 2002). To construct a specific system expressing *GFP* in *M. hyorhinis*, we predicted the *oriC* of *M. hyorhinis* strain HUB-1 (Fig. 1a) following previously methods described in *M. hyoneumoniae* (Maglennon et al. 2013). The *oriC* was PCR amplified from the DNA of *M. hyorhinis* (Fig. 1b) using *oriC* primers listed in Table 1 and was used to replace the *oriC* of *M. hyoneumoniae* in the vector pMD18-TOgfp at *EcoR*I and *Xho*I restriction sites. The resulting plasmid specific for *M. hyorhinis* was designated pMD18-O/MHRgfp. The diagram of the initial cloning and introduction of a new *oriC* is shown in (Fig. 1c). The cloning was verified by restriction enzyme digestion and DNA sequence analysis.

**Fig. 1:**
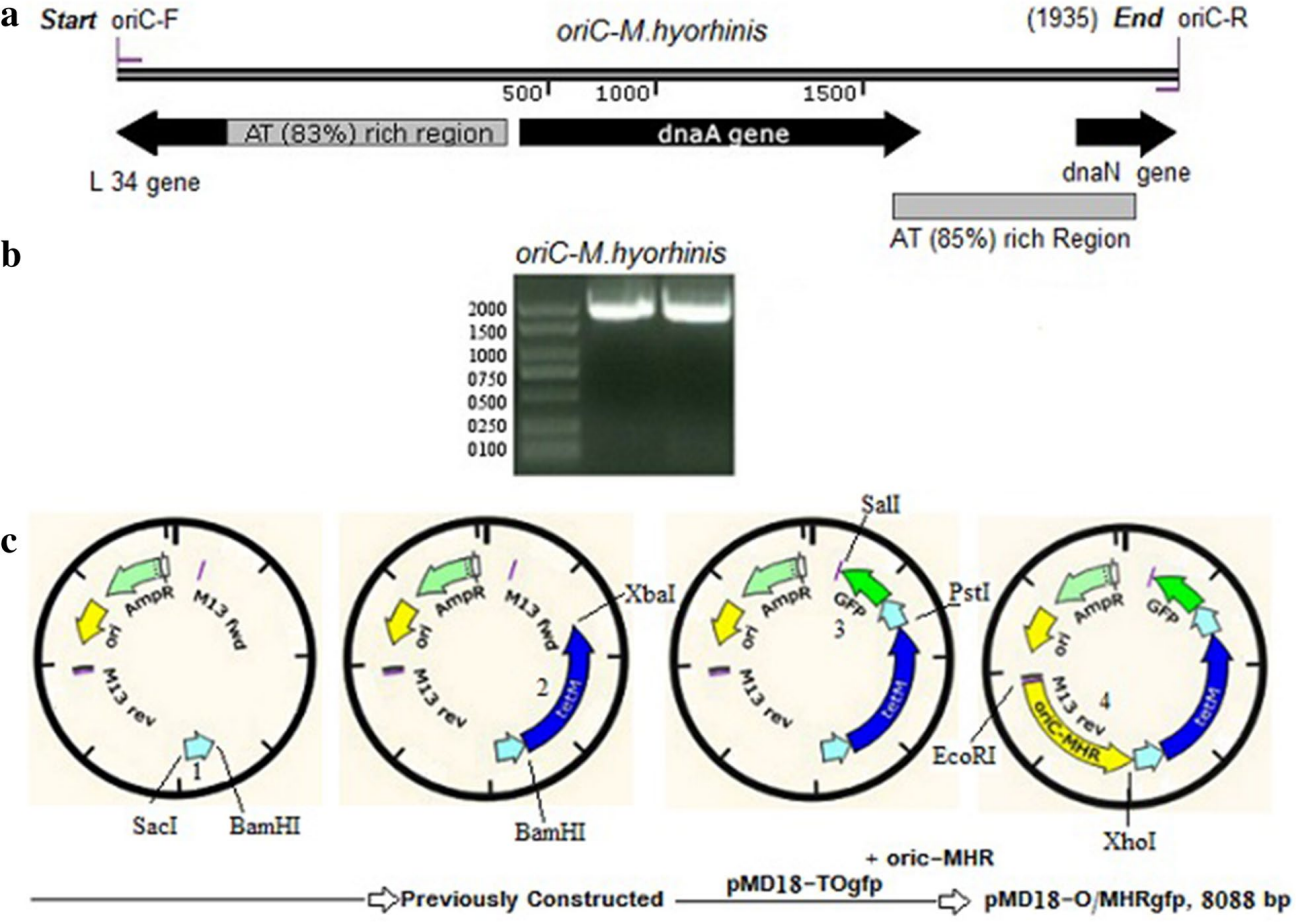
Construction of pMD18-O/MHRgfp. **a** The predicted origin of replication (*oriC*) of *M. hyorhinis* (strain HUB-1). **b** PCR amplified *oriC* fragment (1935 bp). **c** Diagram and map of pMD18-O/MHRgfp construction. The *oriC* of *M. hyopneumoniae* strain 186L was removed from pMD18-TOgfp with *EcoR*I and *Xho*I restriction enzymes and replaced with *oriC* of *M. hyorhinis*. Numbers *1*, *2*, *3* and *4* indicated the order of the fragments cloned into the vector

**Table 1:** Primers used to amplify the *oriC* of *M. hyorhinis* to construct pMD18-O/MHRgfp plasmid

Fragment	Enzymes	Oligonucleotides sequence (5′–3′)	Product (bp)
oriC-MHR	*EcoR*1	Forward: CCGGT**GAATTC**TACCTTTTGCTCTTCTTGCTGCTA	1935
	*Xho*I	Reverse: CGAA**CTCGAG**TAGGAGGATTTCGTCTTATAGG	

Restriction enzyme sites are in bold and underlined

*MHR*, *M. hyorhinis*

Transformation of *M. hyorhinis* by polyethylene glycol (PEG) was reported (Dybvig and Alderete 1988). Here, we optimized methods for transformation by electroporation (Maglennon et al. 2013): We obtained no clones in the KM2-Agar plate containing 0.01 µg/ml tetracycline hydrochloride when we used low voltage (1–1.5 kV) or low concentrations of plasmid DNA (1–5 µg). However, increasing the voltage directly to 2.5 kV and the amount of plasmid DNA to 15 µg could produce 4 × 10^−3^ cfu/µg plasmid DNA. Briefly, 40 ml of *M. hyorhinis* culture were centrifuged at 12,000 rpm for 20 min at 4 °C, and the pellet was washed three times with electroporation buffer (272 mM Sucrose, 200 mM HEPES pH 7.2) supplemented with 1 mM EDTA. The product was incubated on ice for 5 min, and resuspended in 100 µl of electroporation buffer. Plasmid DNA (15 µg) was added to 100 µl competent cells and transferred to chilled 0.2 cm electroporation cuvette (Bio-Rad, USA). The mixture was incubated on ice for 20 min. The cells were electroporated on ECM^®^ 630 Electroporation System, BTX™ at 2.5 kV, 125 Ω and 25 µF. Immediately after electroporation, 900 µl of chilled KM2 medium was added and incubated for 20 min on ice and then recovered for 3 h at 37 °C. The culture was diluted, plated on KM2 plates containing 0.7 % Agar and 0.01 µg/ml of tetracycline hydrochloride and incubated at 37 °C until growth of visible clones. Tetracycline-resistant colonies of transformed mycoplasmas grown on KM2-Agar had appeared within 3–10 days of incubation (Fig. 2a). These colonies were absent in the control mycoplasmas that were not electroporated with plasmid.

**Fig. 2:**
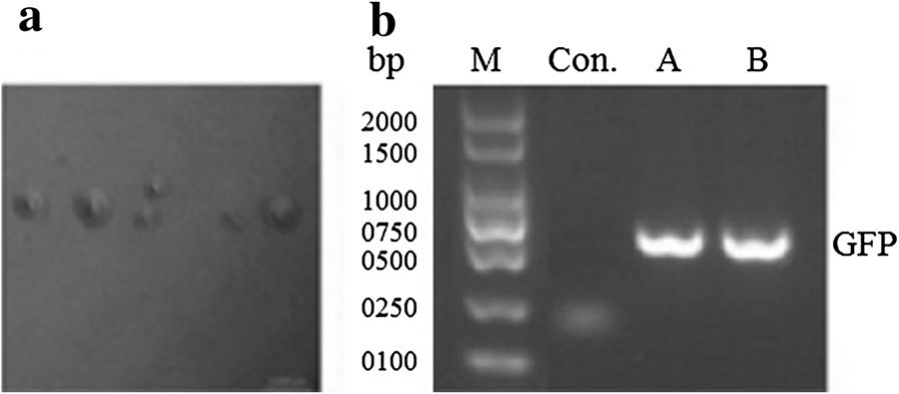
Detection of pMD18-O/MHRgfp in the transformed cells. *M. hyorhinis* were transformed with 15 µg pMD18-O/MHRgfp and grown in KM2 medium containing 0.01 µg/ml tetracycline hydrochloride. The same transformants were also plated in KM2-Agar containing 0.01 µg/ml tetracycline hydrochloride and the plate was incubated for 7 days. **a** Colonies of *M. hyorhinis* observed under microscopy (*scale bar* 100 µM). **b** Transformed mycoplasmas either subcultured in KM2 medium containing 0.01 µg/ml tetracycline hydrochloride or plated in KM2-Agar containing 0.01 µg/ml tetracycline hydrochloride and a single clone picked and sub-cultured in KM2 medium containing 0.01 µg/ml tetracycline hydrochloride. DNA was extracted from control un-transformed mycoplasmas or from transformed mycoplasmas and presence of pMD18-O/MHRgfp was evaluated by PCR to detect *GFP* from KM2 *(b.A)* or from KM2-Agar *(b.B)*. The *GFP* product of about 750 bp was only detected from the DNA of the transformants mycoplasmas and it was consistent with the predicted sizes of *GFP*. This band was absent from the controls (un-transformed mycoplasmas)

Tetracycline-resistant mycoplasmas were analyzed for their plasmid content. Total genomic DNA was extracted using a TIANamp Bacteria DNA Kit (Tiangen, Beijing, China) from either the pool of mycoplasma cultures containing 0.01 µg/ml tetracycline hydrochloride or from a single resistant clone sub-cultured in KM2 medium containing 0.01 µg/ml tetracycline hydrochloride. The presence of pMD18-O/MHRgfp was analyzed by the detection of *GFP* (750 bp) by PCR, and *GFP* could be detected from the total genomic DNA of the transformants, but not from untransformed mycoplasmas (Fig. 2b). One product amplified with *GFP* specific primers was sequenced and was indeed the expected *GFP* sequence (data not shown).

Expression of *GFP* in a single clone of *M. hyorhinis* selected on KM2-Agar was also studied. Seven day-old colonies showed green fluorescence when observed by fluorescence microscopy (Nikon, Eclipse E600, Tokyo, Japan) (Fig. 3) and this color was absent in the controls. The expression of *GFP* in *M. hyorhinis* cells did not appear to interfere with cell growth. Therefore, *GFP* should also be a vital marker of transformation and cell growth as the pure cultures bearing genetic markers can ease the direct identification of cells and colonies among the population of culture. In related studies, the *GFP* gene was described as an efficient marker for studying the development and microbe-plant interaction in the tobacco pathogen *Phytophthoraparasitica* var. *nicotianae* (Bottin et al. 1999). We hypothesize that, tagging *M. hyorhinis* with a plasmid expressing *GFP* may help to follow the infection process by in vivo imaging if *M. hyorhinis* stably harbored the transformed constructs.

**Fig. 3:**
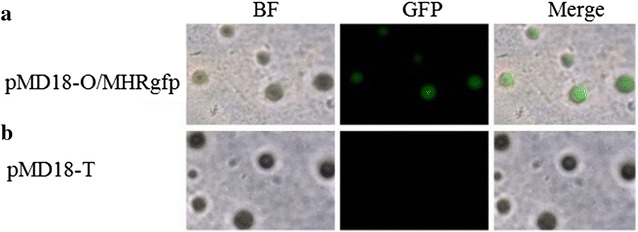
Detection of *GFP* fluorescence. *M. hyorhinis* was transformed with pMD18-O/MHRgfp, plated in KM2-Agar medium containing 0.01 µg/ml tetracycline hydrochloride and cultured for 7 days. Control mycoplasmas were transformed with a pMD18-T empty plasmid. **a** Expression of *GFP* gene upon transformation with pMD18-O/MHRgfp plasmid was observed under the fluorescence microscope. **b** The green fluorescence protein was not observed in the control mycoplasmas transformed with pMD18-T empty plasmid. *Scale bar* 100 µM

In the present report, the construction of a vector carrying the *GFP* gene was performed in order to develop a direct method for monitoring gene transfer and expression in *M. hyorhinis* in which the timing, as well the magnitude of gene expression, is being examined. This visual expression analysis system could also indicate that, the expression of the heterologous genes in *M. hyorhinis* is feasible.
